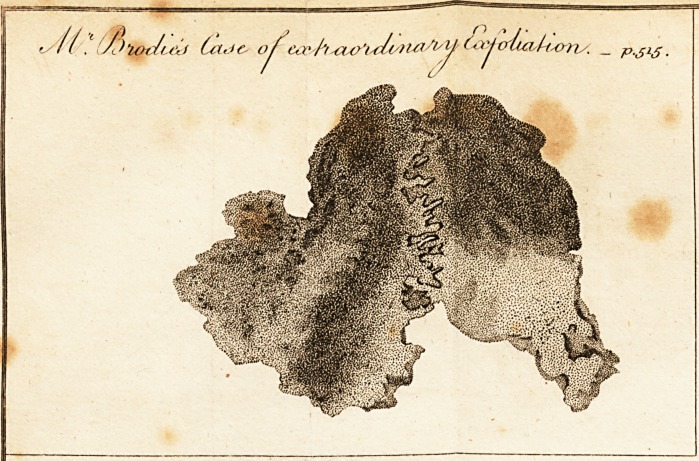# Mr. Brody's Cases in Surgery

**Published:** 1804-12-01

**Authors:** P. W. Brody


### To Dr. GILLESPIE.

Sir,

I Take the liberty of sending you three cases, which I request you will have the goodness to put into a proper form, and if advisable, to publish them through the medium of your learned and respectable friend, Dr. S. Should

---

they be deemed of any importance, it will encourage me to send you occasionally upwards of one hundred and sixty other cases, many of them extraordinary ones. *It has generally been a received opinion that the Indies are unfavourable to surgical operations*, but I can with great truth contradict it, having performed most of the operations we are acquainted with, to the number of one hundred and sixty-five in this Island, and being fortunate enough to lose only six of that number. I have never met a case of tetanus in my practice, but one, which yielded to the cold bath, bark and wine, with opiates occasionally. The *Lobelia* is a new medicine here, and I became possessed of it very accidentally at St. Croix, about eighteen months ago. My friend, Dr. Stevens, formerly a physician of great *eminence, (see Cullen on Digestion.)* had some plants of it sent from South America in a box; I prevailed upon the person who had charge of it, to spare me one small sprig, which, with much care and attention, I have propagated to a considerable extent.

I am, &c.

P. W. BRODY.

---

Cases in Surgery. *By* Mr. Brody, *of Tortola.*

On the 30th of March, a little boy, about eight years of age, belonging to the Honourable Richard Augustus Teahie, had the metatarsal bones of his right foot shattered, and three splinters of wood wedged between them; in this situation a medical gentleman, who in my absence attended for me, wrapped the foot up, after applying lint and spirit to the laceration. On the following day I had the boy brought to my Hospital; and upon opening the wound and introducing my finger, found splinters, which with some difficulty I extracted, as well as several pieces of bone. The boy had passed a restless night, though an opiate had been given; he had a smart fever and slight delirium. I ordered an aperient mixture, which produced two good evacuations. At noon, the fever still continued, and the parts looked very livid; I took off his leg at the usual place, and upon unscrewing the tourniquet, to my great surprise, not a drop of blood issued from the arteries. I gave him bumpers of Madiera wine, and waited in vain for half an hour in expectation of seeing the arteries, in order to secure them, but could not. He continued feverish for two days after the operation, during which time he took the saline mixture; his body was kept soluble soluble by injections, castor oil, and a decoction of manna and senna; he had large anodynes at night, but rested indifferently. He could retain no nourishment until the 3d of April. When the dressings were removed, they came away with great ease, and the stump looked healthy and in every respect as well as most I have seen; his fever now left him, his appetite returned, and he continued taking bark and nourishment freely until the 10th of May, when he was discharged cured.

What could prevent the effusion of blood? He lost very little at the time of the accident. He was under no apprehension at the time of the operation; indeed, he was in a state of delirium.

I have since had an operation where the patient died on the 3d day after it. He was an athletic man, about twenty-five years of age, and was wounded on board a privateer belonging to Mr. J. Dougan, the Navy Agent here; from the time he received the wound, which was in his wrist, he became delirious, without fever, and continued so until his death. I had his head shaved and minutely examined, but could trace no sign of injury.

I have sent you a drawing of an extraordinary exfoliation. *(See plate.)* The subject was a Dutchman, aged twenty-five, and sent to me in November last from H. M. ship, Chichester, Capt. Spears, who received him from Barbadoes Hospital, where he had been invalided. I understood from himself he had been sent to the hospital for typhus, and that his head had been blistered, and the integuments sloughed off from all the fore-part. When brought here he was a most dismal object, his sore resembling a cancerous one; wherever any of the corrosive discharge touched, it excoriated and induced a sordid ulcer. I gave him bark, wine, anodynes, &c. I washed the ulcer with a decoction of cicuta, and administered the extract internally to no purpose for two months; the good nourishment and healthy situation proved in some degree serviceable, as his appetite became better and strength amended. About this period I understood he had been afflicted with syphilis about eighteen months ago, which induced me to put him upon a slight mercurial course, the ungt. hydrarg. fort. was used ℥ij. every night for three weeks, without producing any effect upon his mouth; the sore still looking very ill with glossy surface full of small holes, from which issued a most fœtid and caustic discharge; at this time the black part of the drawiug was quite bare. Being still of opinion that syphilitic virus was the principal impediment to the cure, I had recourse to a strong decoction of the lobelia (which I have propagated largely in my garden); he took about a quart of it daily. In ten days the wound looked better, the bone became loose, except at the angles, where I was obliged to use the knife to disengage it. About the size of a half crown piece of the dura mater was bare, which I covered with soft lint every morning and evening for about a fortnight, when granulations were formed and the part filled up apace; from that time his health returned, and he was perfectly cured and sent home in the Ulysses.

Remark.—The lobelia may be found to prove a medicine of efficacy in various other complaints as well as in syphilis; the good effects therefore of this simple, in the present case, is far from establishing the identity of syphilitic disease in this instance.

## Figures and Tables

**Figure f1:**